# Supplementary material for: Causal Relationship between Aging and Anorexia Nervosa: A White-Matter-Microstructure-Mediated Mendelian Randomization Analysis
Source: Biomedicines. 2024 Aug 16;12(8):1874. doi: 10.3390/biomedicines12081874 (PMC11351342; doi:10.3390/biomedicines12081874)
Supplement: Supplementary file 1 [file biomedicines-12-01874-s001.zip › Supplementary Figure/Figure S9/Leaveoneout plot of MR analysis for ubm-b-1886 to AN.pdf]

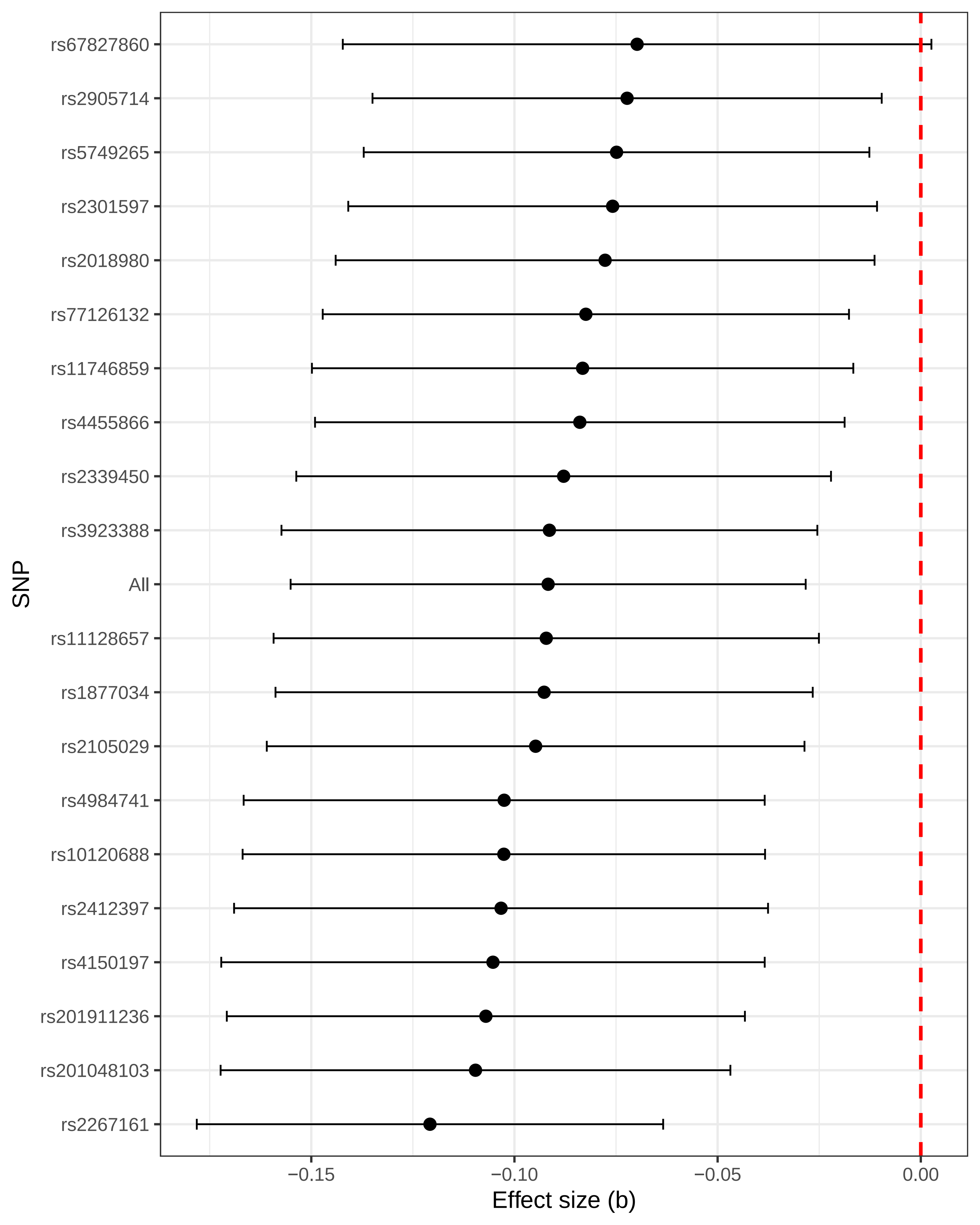

SNP
rs67827860
rs2905714
rs5749265
rs2301597
rs2018980
rs77126132
rs11746859
rs4455866
rs2339450
rs3923388
All
rs11128657
rs1877034
rs2105029
rs4984741
rs10120688
rs2412397
rs4150197
rs201911236
rs201048103
rs2267161
−0.15
−0.10
−0.05
0.00
Effect size (b)
